# Enhancing Allocentric Spatial Recall in Pre-schoolers through Navigational Training Programme

**DOI:** 10.3389/fnins.2017.00574

**Published:** 2017-10-16

**Authors:** Maddalena Boccia, Michela Rosella, Francesca Vecchione, Antonio Tanzilli, Liana Palermo, Simonetta D'Amico, Cecilia Guariglia, Laura Piccardi

**Affiliations:** ^1^Neuropsychology Unit, IRCCS Fondazione Santa Lucia of Rome, Rome, Italy; ^2^Department of Psychology, Sapienza Università di Roma, Rome, Italy; ^3^Life, Health and Environmental Science Department, University of L'Aquila, L'Aquila, Italy; ^4^Department of Medical and Surgical Sciences, University Magna Graecia, Catanzaro, Italy; ^5^Department of Biotechnological and Applied Clinical Science, University of L'Aquila, L'Aquila, Italy

**Keywords:** human navigation, normal development, allocentric representation, egocentric representation, spatial orientation training, environmental knowledge, survey knowledge

## Abstract

Unlike for other abilities, children do not receive systematic spatial orientation training at school, even though navigational training during adulthood improves spatial skills. We investigated whether navigational training programme (NTP) improved spatial orientation skills in pre-schoolers. We administered 12-week NTP to seventeen 4- to 5-year-old children (training group, TG). The TG children and 17 age-matched children (control group, CG) who underwent standard didactics were tested twice before (T_0_) and after (T_1_) the NTP using tasks that tap into landmark, route and survey representations. We determined that the TG participants significantly improved their performances in the most demanding navigational task, which is the task that taps into survey representation. This improvement was significantly higher than that observed in the CG, suggesting that NTP fostered the acquisition of survey representation. Such representation is typically achieved by age seven. This finding suggests that NTP improves performance on higher-level navigational tasks in pre-schoolers.

## Introduction

Representing and transforming spatial information are everyday activities that are crucial to moving to a new town, reading and interpreting maps. Human navigation requires the ability to mentally transform images from two-dimensional to three-dimensional forms, similar to following a map to reach a goal in a new environment. It also requires recognizing a place from a different perspective and finding an alternative route when an initial route is interrupted. Navigational ability and spatial behavior develop gradually and at distinct time points during childhood (Siegel and White, 1975; Lehnung et al., 2003). According to Siegel and White's model 1975, environmental knowledge is acquired in three separate and distinct steps: (i) *landmark knowledge*, with which individuals are able to perceptually discriminate and recognize landmarks but are unable to derive directional information from them (the location of a landmark, its relation with the environment and its relation to other landmarks); (ii) *route knowledge*, with which directional information based on egocentric representation is added to landmark knowledge, allowing individuals to navigate by following directional instructions that link consecutive landmarks (e.g., turn right at the bakery to reach the theater); and (iii) *survey knowledge*, with which individuals build a mental map of the environment based on an allocentric frame of reference. The way in which environmental knowledge is acquired, as well as its organization continue to be debated. On the one hand, Siegel and White's Model suggests a hierarchical organization in which phases are acquired sequentially (Siegel and White, 1975). On the other hand, Montello (1998) suggests an environmental representation acquired simultaneously. One must distinguish between two different frames of reference in environmental mental representation, namely, egocentric and allocentric representations of the environment. *Egocentric representation* expresses the relation of an environmental object with respect to the self, and this frame of reference is generated from sensory data and may provide a direct basis for action; *allocentric representation* expresses the location of the environmental object with respect to an external frame of reference, which is more difficult to compute but provides a better basis for flexible navigation and long-term storage of complex layouts (Byrne et al., 2007). Egocentric and allocentric representations roughly correspond to the route and survey knowledge described in Siegel and White's Model. In route-based navigation, individuals use egocentric coordinates, whereas in survey-based navigation, they mainly use allocentric coordinates. The nature of spatial representation in the brain indicates the parallel presence of both survey and route knowledge. Individuals could proficiently shift from a route to a survey perspective (Taylor and Tversky, 1992), even if the environment has been learned using the other perspective (Boccia et al., 2016a). Neural activity within the brain network underlying human navigation, such as the parahippocampal place area (PPA) and the retrosplenial cortex (RSC) (Boccia et al., 2014, 2016b), has been found to depend on the familiarization and the type of strategy participants adopt in performing a topographical memory task (Boccia et al., 2016a). These findings suggest that these brain areas underlie the shift between different types of environmental knowledge, namely, route and survey knowledge. This result confirms the idea that environmental objects are processed in parallel in different formats and that a proficient shift from one format to another may occur from the first stage of environmental knowledge acquisition (Montello, 1998).

Regarding the development of navigational abilities during childhood, some competences are developed during the early years, while others require a longer time to become fully functioning. Overall, studies suggest that although some spatial knowledge may be innate, the majority of skills requires time to fully mature (Vasilyeva and Lourenco, 2012; Nys et al., 2015). Some of these skills develop early in life, such as the use of egocentric strategies to find a hidden target, reference memory and visuo-spatial working memory (Piaget and Inhelder, 1948; Acredolo, 1978; Bremner, 1978; Acredolo and Evans, 1980; Foreman et al., 1984, 1990), while others require a longer period to fully develop. For example, the development of navigational working memory is complete at ~10 years of age (Piccardi et al., 2014b). A similar developmental trajectory holds for the ability to construct a cognitive map of the environment based on distal cues, that is, a stable mental representation of navigational space (O'Keefe and Nadel, 1979). This competence becomes fully developed at approximately only 7 years of age (Overman et al., 1996; Lehnung et al., 1998, 2003). Later, by age 10, relational place strategies, which are necessary for cognitive mapping, develop (Overman et al., 1996; Lehnung et al., 1998). More recent studies suggest that by the age of 4, children use movement information or unique proximal landmarks to solve a viewpoint-independent reorientation task in which spatial recall cannot be performed using a stored view, that is, using a flexible representation of the environmental layout. Instead, a flexible recall from novel viewpoints is available by the ages of 6 to 8 (Nardini et al., 2009). Interestingly, 4-year-old children have some basic features of allocentric coding (Negen et al., 2017). Moreover, the spontaneous use of an allocentric world-centered representation of the environment progressively increases between 5 and 10 years of age (Bullens et al., 2010), even if younger children are able to use an allocentric strategy when aided (Bullens et al., 2010). Overall, these findings suggest that a “developmental window” occurs between 4 and 8 years of age and poses a new and fascinating question regarding whether specific formal training may foster navigational skills in children.

However, compared to other human skills (such as math, reading, writing, and problem solving) that receive formal training during childhood, spatial orientation is not strengthened through specific educational training at school, during childhood or later, with the exception of individuals trained to use this competence at higher levels in a professional context, e.g., aerospace pilots, astronauts, medical surgeons, topographers, or military raiders (Apuzzo, 1996; Verde et al., 2013, 2015, 2016). It is largely accepted that learning produces a brain re-organization that acts as a “brain reserve,” increasing the brain's tolerance to disease (Stern, 2012; Colangeli et al., 2016). It has been shown that the brain may be continuously modified by life experiences (Verde et al., 2013, 2015, 2016), even when the brain is affected by neuropathology or when cognitive training enhances the effect of pharmacological therapy (Onder et al., 2005). For this reason, navigational training in pre-schoolers may foster navigational ability and prevent the development of navigational disorders, such as developmental topographical disorientation (DTD: Iaria et al., 2005, 2009; Bianchini et al., 2010). DTD is a developmental disorder for which the etiology is currently unknown; no cerebral damage or psychiatric disorders have been associated with it. Iaria and Barton (2010) showed that DTD is widespread in the population, and the detailed descriptions of healthy individuals who suffer from DTD (refer to Bianchini et al., 2014b; Palermo et al., 2014; Nemmi et al., 2015; Piccardi et al., under revision) appear to suggest that this neurodevelopmental disorder should be monitored from childhood to prevent the persistence of the disorder in adults. The existence of DTD and its different types strongly suggests the importance of introducing navigational training during early infancy. Furthermore, navigational training may be useful for blind children. Research appears to suggest that children with visual impairments tend to rely on egocentric encoding (Ruggiero et al., 2009; Iossifova and Marmolejo-Ramos, 2013). Moreover, visually impaired children show a tendency for narrowing spatial frames by substituting the allocentric space with egocentric or bodily space (Iossifova and Marmolejo-Ramos, 2013). These findings appear to suggest that a lack of vision may affect allocentric but not egocentric frames of references.

**Figure 1:**
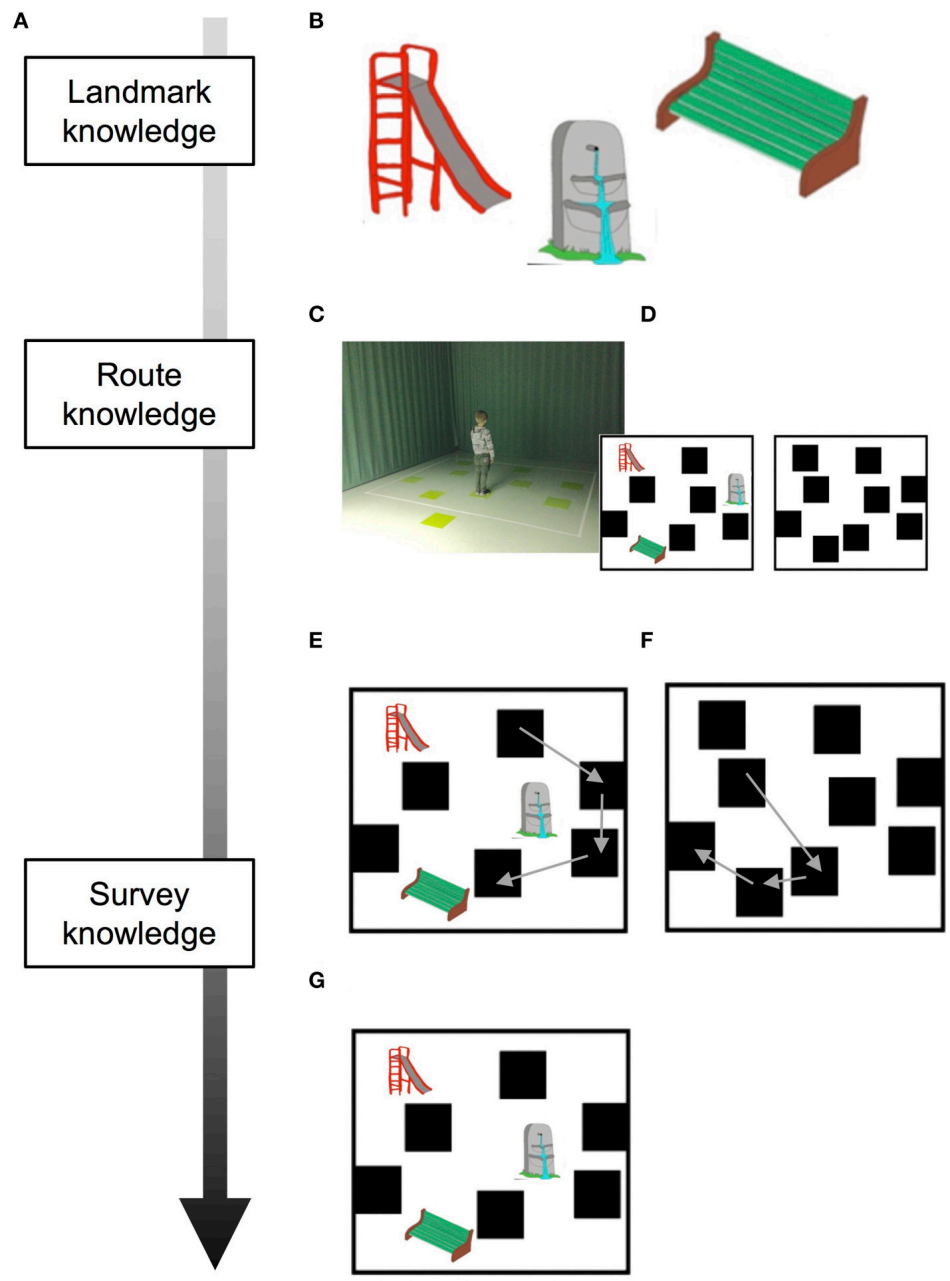
Schematic depiction of tasks and results according to the stages of acquisition of spatial knowledge. **(A)** Siegel and White's model of acquisition of spatial knowledge. **(B)** Landmarks used during WalCT. **(C)** WalCT apparatus; **(D)** Sketch maps of the WalCT with and without landmarks are represented on the bottom-left and -right, respectively. **(E)** Drawing of the pathway in the L-WalCT and **(F)** the WL-WalCT outlines. **(G)** Landmark location on the map. Written informed consent was obtained from both parents of the child represented in the figure for the publication of this study.

Here, we aimed to investigate the effectiveness of navigational training programme (NTP) to develop spatial orientation skills in 4-year-old children based on Siegel and White's hierarchical model. According to the aforementioned studies, we expected that this early period of life may constitute a developmental window in which it is possible to observe training effects. With this aim, we administered a 12-week NTP and tested the children in both the training and control groups twice (before and after NTP) on navigational tasks that tap into landmark, route and survey knowledge (Figure 1A). We hypothesized that the NTP would yield a better performance on highly demanding navigational tasks (i.e., survey knowledge-based tasks; Figure 1A) in the training group (TG) than that seen in the control group, which was not exposed to the training. A comparison of these groups enabled us to subtract the normal development of these skills from the improvement produced by the NTP. We expected an earlier acquisition of the ability to form a cognitive map of the environment to be detected only in the group exposed to NTP.

## Materials and methodology

### Participants

The present study was conducted in a sample of 34 typically developing Italian children (19 M and 15 F) who were recruited from a primary school, the “Istituto Comprensivo di Via Anagni,” in Rome (Italy). Ages ranged from 49 to 72 months (mean age = 63.09 months; SD = 2.12 months).

None of the children included in the study had primary visual or hearing impairments, had been diagnosed with a neurological condition or had ever exhibited emotional or behavioral problems. To determine their general cognitive level, all children were administered Raven's Colored Progressive Matrices (Raven, 1986; Belacchi et al., 2008); no difficulty in clear-thinking ability emerged. The study was approved by the local ethics committee of the Psychology Department of “Sapienza” University of Rome in accordance with the Declaration of Helsinki. Written and informed consent was obtained from the parents of each child. The children were subdivided into two groups consisting of 17 participants per group and were comparable in age [*t*_(32)_ = 0.08; *p* = 0.93] and gender [chi-squared = 28.94; *p* = 0.62].

### Instruments and procedure

The children were tested individually in a quiet room in kindergarten. The TG was tested immediately before and after the NTP period at T_0_ and T_1_, respectively. The control group (CG), which received standard didactics, was tested at T_0_ and T_1_ without being subjected to a training procedure.

### Training procedure

NTP was conceived to enhance the visuo-spatial abilities that underlie human navigation, such as mental rotation (Piccardi et al., 2017), visuo-spatial and navigational memory (Piccardi and Nori, 2011), navigational planning (Bocchi et al., 2017), spatial orientation, left and right discrimination and spatial representation of the body according to Siegel and White's hierarchical model. Each activity fosters a specific level of the acquisition of navigational knowledge, namely, landmark, route, and survey knowledge. Thus, activities involving landmark and pictorial recognition and perceptual discrimination were thought to foster landmark representation; activities involving the acquisition of directional egocentric information were thought to foster the acquisition of route representation; finally, activities that targeted mental transformation and map-based orientation were thought to foster the acquisition of survey representation (see the description of activities below for details). The activities were administered according to the hierarchical model with increasing levels of difficulty. The NTP was administered at the school and covered 18 sessions (1.5 h each) during a period of 12 weeks (see Supplementary Figure 1A for the experimental timeline). The protocol comprised paper-and-pencil and navigational activities (see the detailed description of activities below). The experimenter ascertained that all children played together during navigational activities. Each session was structured to contain both types of activities. Most procedures were inspired by Piccardi (2011) and are subsequently described below according to the level they fostered.

### Landmark knowledge acquisition

#### Colored matrix (visuo-spatial memory)

This task is a paper-and-pencil activity. The children were provided with a set of colored paper-and-pencil matrices (Supplementary Figure 1B) presented one by one in the center of a vertically aligned sheet of white paper (A4 format). In each matrix, boxes were only partially filled-in. The children were instructed to observe the colored boxes in the matrix. After 1 min of observation, the children were required to turn the page and fill-in the same boxes in an empty matrix. This task included seven trials, with increasing levels of difficulty.

#### Large-scale memory

This task is a practical spatial game. We developed a larger version of the classic “memory game,” the card game in which all cards are placed face down on a surface and only two cards may be turned face up during each turn. The object of the game is to turn over all pairs of matching cards. In the large-scale memory exercise, we used 10 pairs of pictures representing different animals (i.e., sheep, elephant, pig, crocodile), which were manually drawn by one of the authors (LPi) on sheets of white paper (A4 format) and were vertically placed on the floor of a large room covering a surface of 2.5 × 2.5 m. The children were divided into two teams. All participants on each team turned over a pair of sheets in turn. When a participant found the matching animal, the participant took both sheets, and another participant on the same team turned over another pair of sheets. When a participant failed to obtain a match, another participant attempted to obtain a match. The team with the highest number of matching sheets won the game.

### Route knowledge acquisition

#### Paper-and-pencil Labyrinth (navigational planning)

This task is a paper-and-pencil activity. The children were provided with a set of pictures, including (a) a subject (a child or an animal), (b) a goal target (e.g., food, water), and (c) a path between the subject and the goal target (Supplementary Figure 1C). They were instructed to draw the path the subject had to follow to reach his/her goal. For the first 8 trials, the path was unambiguous, whereas the last 3 trials showed a high level of complexity and some dead ends.

#### Joystick (spatial orientation and left/right discrimination)

This task is a practical spatial game. A leader was selected among the children. All other children were required to place themselves in front of the leader. The leader verbally instructed the participants to move forward, backward, to the left, or to the right. He/she could give the instruction more or less quickly in order to make it more difficult for them participants to follow his/her instructions. The participants who got the order wrong were out of the game. The last player left became the leader of the following game session.

#### The navigator (spatial orientation and left/right discrimination)

This task is a practical spatial game. The players were divided into pairs comprising one player guiding (i.e., the guide) and the other navigating blindfolded (i.e., the navigator). At the end of each turn, the players traded places. A guide was chosen, and he/she hid an object. Each guide had to guide his/her blindfolded navigator to be the first to find the hidden object. Only directional (e.g., right, left) and numerical (e.g., 2 steps, 3 steps) cues were allowed. The first pair to find the object could hide it during the following turn and decide which pair should have the blindfolded navigator.

#### Paths (spatial orientation)

This task is a practical spatial game. The children were divided into teams. The teacher built a path, and each team, in turn, had to follow it. The team that completed the path the fastest won the game.

#### Juggle (spatial representation of the body)

This task is a practical spatial game. Each child received a balloon to juggle. The first children were allowed to juggle with any part of the body. After this initial phase, the children were instructed to juggle with a specific part of the body (e.g., the right hand, the left foot). The child who kept the balloon for the longest time won the game.

#### Up and down (spatial representation of the body)

This task is a practical spatial game. The children were divided into two teams. The players on each team formed a line, and the first player of each line had a balloon. At a starting signal, the player with the balloon gave it to the player behind him/her by passing it over his/her head. The second player passed the balloon to the third player by passing it under his/her legs. The third player passed the balloon to the fourth player by passing it over his/her head and so on to the last player in the line. The team whose balloon reached the last player first won the game.

#### In and out of the hoop (spatial representation of the body)

This task is a practical spatial game. The children were divided into two teams. The players of each team formed a line at ~2 m from the other team. A hoop was given to the first player of the line. At a starting signal, the player stepped into the hoop with his/her foot and lifted it up over his/her head. The player behind him/her grabbed the hoop and did the same, as did the other players until the last player in the line. The team that reached the last player first won the game.

### Survey knowledge acquisition

#### Objects' mental rotation (mental rotation)

This task is a paper-and-pencil activity. The children were provided with a set of 10 sheets where (a) a target flower in the center of the sheet and (b) three test flowers below the target item (Supplementary Figure 1D) were depicted. Only one of the three test flowers corresponded to the target. The children were required to find the correct flower among the test flowers.

#### The explorers (spatial orientation and navigational memory)

This task is a practical spatial game. The children were divided into teams or small groups and were invited to navigate through the school (e.g., visiting the garden) for ~15 min and register as many details (e.g., sounds, odors, objects, and positions) as possible. When they returned to the classroom, they were instructed to draw a map of the path they had taken. They were also required to answer questions regarding the details on their map. The team that drew the best map and answered the most questions won the game.

#### Testing procedures

We tested verbal comprehension of spatial locatives using the Test for Reception of Grammar (TROG: Bishop, 1982; Italian version: Chilosi and Cipriani, 1995), which assesses grammatical comprehension from age 4 to adulthood. Each test stimulus is presented in a four-picture, multiple-choice format with lexical and grammatical foils. The grammatical complexity increases consistently from locative structure to active, passive, negative, dative and relative clauses. For experimental purposes, we selected only spatial locative sentences (14 sentences); these sentences included locative topological elements (below/above, up/down, in/out and near/far) and prospective locative elements (in front of/behind, from/to and between). The participants' task was to select the picture that matched a sentence spoken by the examiner; in the case of errors, the sentence was repeated. The score was 0 if the answer was immediately correct, 0.5 if the answer was correct after repetition and 1 if the answer was wrong.

Topographical learning (TL) was assessed using the Walking Corsi Test (WalCT: Piccardi et al., 2008, 2014a,b). The WalCT is a larger version (3 × 2.5 m; scale 1:10) of the Corsi Block Tapping Test (CBT; Corsi, 1972). It has been used for experimental and clinical purposes (Piccardi et al., 2008, 2014a,b; Bianchini et al., 2010, 2014a,b; Nemmi et al., 2013; Palermo et al., 2014) to investigate topographical memory by instructing individuals to reproduce a previously observed pathway. The WalCT is set up in an empty room. It is composed of nine black squares (30 × 30 cm) placed on the floor. This test is scaled to the standard CBT. The starting point is the black square located outside the layout.

In the WalCT, the examiner walks and stops on a series of squares. The subject must walk, reach different locations and reproduce the sequence demonstrated by the examiner. In this study, two aspects of topographical long-term memory were assessed, based on the results of a previous study (Piccardi et al., 2015): TL and topographical delayed recall (TDR) under two different conditions, namely, with landmarks (L-WalCT) and without landmarks (WL-WalCT) (Figures 1B–D). The only difference between the L-WalCT and WL-WalCT conditions was the presence in the L-WalCT of pictures of three landmarks placed on three black squares (Figure 1B).

In the TL, the children had to learn a fixed supra-span sequence, which was calculated according to their chronological age by considering the span plus 2 according to the median span sample of Piccardi et al.'s (2014b) study. Specifically, 4-year-old children had to learn a 4-block sequence (because the median span was 1.90 at this age). At each trial, after the examiner presented the sequence, the child was invited to step onto the carpet to reproduce it and step off the carpet when he/she had finished (Figures 1C–D). In each trial, the number of correct black squares reproduced in the sequence was calculated for the final score, but no feedback regarding performance correctness was provided. The learning criterion (indicating that learning was achieved) corresponds to three consecutive correct sequence reproductions; if the child did not achieve the learning criterion, the sequence was repeated for a maximum of 18 trials. The learning score was calculated by attributing one point for each square correctly walked until the criterion was achieved; the score corresponding to the correct performance of the remaining trials was added to this score (up to the 18th; maximum score: 72). After 5 min, the TDR was administered. The examiner instructed each participant to reproduce the previously learned four-block sequence in a single attempt. The score represented the number of squares correctly reproduced. TL and TDR may be considered as tapping into *route knowledge* (Siegel and White, 1975).

At the end of the L-WalCT, the children were shown 6 items on an A4 sheet (3 landmarks and 3 distractors). They had to indicate which of the landmarks were present in the L-WalCT (landmark recognition). An individual's score corresponded to the sum of correct responses (maximum score: 6). This task may be considered as tapping into *landmark knowledge* (Siegel and White, 1975). The children were subsequently instructed to place small pictures depicting 3 landmarks on an outline of the WalCT as they experienced them during the administration of the WalCT (landmark location). In this case, an individual's score also corresponded to the sum of correct responses (maximum score: 3) (Figure 1G). This task may be considered as tapping into *survey knowledge* (Siegel and White, 1975). At the end of both the L-WalCT and WalCT, the children were also instructed to use a felt-tip marker to retrace the pathways they had learned during the two conditions on the outline of the WalCT. An individual's score corresponded to the sum of the squares correctly identified during the line-tracing (drawing; maximum score: 4) (Figures 1E,F). The path line retracing in the drawing of both the L-WalCT and WL-WalCT may be considered the transposition of route knowledge on a map-like/survey representation of the environment and thus an intermediate stage between route- and survey-based knowledge representation (for a schematic depiction of tasks and knowledge representation, see Figures 1A,E,F).

### Statistical analysis

For analysis, 2 × 2 mixed factorial ANOVAs with Group (CT and TG) as the between factor and time (T_0_ and T_1_) as the repeated measures were performed to detect the effect of training on (1) spatial locative comprehension (the number of errors on the TROG), (2) landmark recognition, (3) TL in the L-WalCT, (4) TL in the WL-WalCT, (5) the TDR in the L-WalCT, (6) the TDR in the WL-WalCT, (7) the drawing of the path line on the L-WalCT and (8) on the WL-WalCT outline, and (9) landmark location on the map. The alpha level was set at *p* = 0.05. Eta effect sizes (η2) were computed for main and interaction effects. The benchmarks available to interpret η2 are 0.01–small, 0.06–medium, and 0.14–large (Kittler et al., 2007). However, these benchmarks have not been previously indicated for psychological data (as highlighted by Iossifova and Marmolejo-Ramos, 2013). Therefore, “as η can refer to linear and non-linear relationships, η can be considered a general case in which r is a special example (Rosenthal and Rosnow, 2008). Thus, the generally accepted regression benchmark for effect size r can be used to interpret η: small–0.10, medium–0.30, and large–0.50 (Cohen, 1992)” (p. 2178 in Iossifova and Marmolejo-Ramos, 2013). *Post-hoc* comparisons were performed by applying Bonferroni's correction for multiple comparisons.

## Results

The children participated in the training sessions an average of 71.24% (SD = 12.92) of the “maximum” training time.

We identified a main effect of time on spatial locative comprehension as measured by the TROG [*F*_(1, 32)_ = 17.89; *p* < 0.001; Partial Eta Squared = 0.36], with better performances at T_1_ (Table 1). No other effect was identified on the scores of the TROG.

**Table 1:** Means and SDs of the experimental tasks.

**Task**	**T**_**0**_		**T**_**1**_	
	**CG**	**TG**	**CG**	**TG**
TROG	2.09 (1.63)	2.91 (2.31)	0.618 (1.28)	1.29 (1.25)
**LANDMARK KNOWLEDGE-BASED TASK**				
Landmark recognition	5.94 (0.24)	5.94 (0.24)	6 (0)	6 (0)
**ROUTE KNOWLEDGE-BASED TASKS**				
TL, L-WalCT	68.41 (6.29)	69.12 (6.59)	71.29 (1.40)	71.71 (0.85)
TL, WL-WalCT	65.24 (9.47)	68.94 (6.14)	69.71 (2.64)	70.12 (3.82)
TDR, L-WalCT	3.88 (0.49)	4 (0)	3.88 (0.49)	4 (0)
TDR, WL-WalCT	3.76 (0.97)	3.71 (0.77)	4 (0)	4 (0)
**ROUTE/SURVEY KNOWLEDGE-BASED TASKS**				
Drawing L-WalCT	2.76 (1.52)	2.06 (1.60)	2.94 (1.60)	3.53 (0.87)
Drawing WL-WalCT	2.65 (1.54)	2.35 (1.54)	3.24 (1.35)	3.65 (0.79)
**SURVEY KNOWLEDGE-BASED TASK**				
Landmark location	2.12 (1.05)	1.76 (1.15)	1.88 (1.27)	2.71 (0.85)

*TL, topographical learning; TDR, topographical delayed recall; L, with landmarks; WL, without landmarks; WalCT, Walking Corsi Test; TROG, Test for Reception of Grammar (errors)*.

A main effect of time was also identified for the TL in the L-WalCT [*F*_(1, 32)_ = 6.93; *p* = 0.01; Partial Eta Squared = 0.18] with a similar effect in the WL-WalCT [*F*_(1, 32)_ = 4.06; *p* = 0.05; Partial Eta Squared = 0.11]. For both tasks, performances were better at T_1_ (Table 1).

**Figure 2:**
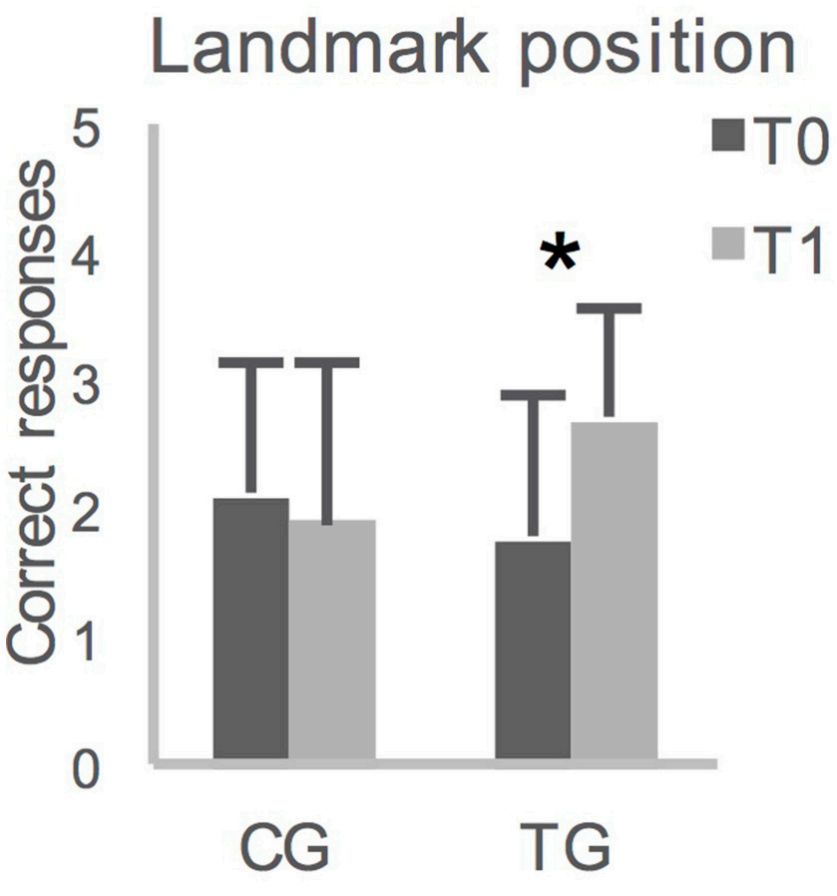
Averaged group performances (and standard deviations) on Landmark location on the map, before (T_0_) and after (T_1_) 12-weeks. CG, control group; TG, training group; ^*^p = 0.007 with Bonferroni's correction for multiple comparisons.

The ANOVA on performances in the landmark recognition (Table 1), as well as the ANOVAs on the TDR in the L-WalCT and the WL-WalCT (Table 1), showed no significant effects. The ANOVAs on the drawing of the path line showed a main effect of time both in the L-WalCT [*F*_(1, 32)_ = 4.77; *p* = 0.04; Partial Eta Squared = 0.13] and the WL-WalCT outline [*F*_(1, 32)_ = 10.29; *p* < 0.01; Partial Eta Squared = 0.24], with better performances at T_1_ (Table 1). Interestingly, the ANOVA on landmark location performances showed a group by time interaction effect [*F*_(1, 32)_ = 6.49; *p* = 0.02; Partial Eta Squared = 0.17]. *Post-hoc* comparisons showed that the two groups significantly differed at T_1_ (*p* = 0.03 Bonferroni's correction for multiple comparisons), with the TG performing better than the CG (Table 1). Moreover, the children in the TG significantly ameliorated their performances at T_1_ compared with that at T_0_ (Figure 2).

## Discussion

We hypothesized that navigational training during childhood may promote an earlier acquisition of high level spatial abilities. Unlike many other abilities, which receive educational training at school during childhood, spatial orientation does not receive systematic training. Nevertheless, high levels of navigational training in adulthood significantly improve spatial orientation ability (Verde et al., 2013, 2015, 2016). The present results show that NTP enhanced the ability to transform egocentric navigational information in a map-like, allocentric representation of the environment in 4- to 5-year old children.

As previously discussed, even if 4-year-old children have basic features of allocentric coding (Negen et al., 2017), children typically master this ability at ~7 years of age. Spatial orientation abilities are fully functioning by age 10, with an increase in the spontaneous use of allocentric world-centered representation of the environment (Bullens et al., 2010). Similarly, in this study, we found that children (mean 63.09 ± 2.12 months) who underwent NTP were more proficient in locating landmarks on a map than their peers who did not receive undergo NTP. This result suggests that NTP yields an earlier development of an allocentric world-centered representation of the environment. To locate landmarks on a map, children must translate egocentric tri-dimensional information regarding the environment they experience in an allocentric bi-dimensional representation. This operation is similar to a “cognitive map,” that is, a stable mental spatial representation independent of perception (Tolman, 1948; Boccia et al., 2017). When required to locate landmarks on a map, children must retrieve navigational information regarding their position from memory and convert it into a map-like representation. As a result of NTP, the children developed the ability to use allocentric coding earlier, and they could pinpoint a location in relation to other locations rather than using egocentric coding. The use of allocentric coding requires an individual to generate, maintain, inspect and transform an image in the mind. These aspects of mental imagery (Kosslyn, 1980) are pivotal to human navigation (Pazzaglia and De Beni, 2006; Palermo et al., 2012; Piccardi et al., 2017). The NTP encompassed activities involving mental rotation (i.e., mental transformation) and visuo-spatial memory (i.e., maintaining an online mental image), as well as activities focused on spatial orientation, left/right discrimination, navigational memory, and planning. The finding that the TG achieved the ability to use an allocentric coding earlier than the CG suggests that a formal training of visuo-spatial abilities that underlie human navigation (such as mental rotation, visualization, or navigational problem solving) improves the ability to transform egocentric representation into an allocentric representation.

Interestingly, our data suggest that the development of spatial locative comprehension and mastery of route knowledge-based tasks (i.e., L-WalCT and WL-WalCT) and the path-line drawing on a map (i.e., Drawing L-WalCT and Drawing WL-WalCT) spontaneously improved from T_0_ to T_1_ in the CG as well. In contrast, landmark location on the map is the only task significantly improved by NTP. Route knowledge-based tasks require only an egocentric frame of reference, which may also be used during a task that requires drawing a path. Individuals who have not fully developed an allocentric strategy may trace the line of the path by simply repeating the egocentric path they learned. Instead, when individuals are asked to locate landmarks on a map, they must use both egocentric and allocentric information. Thus, even if performances on route knowledge-based tasks spontaneously increase in 4- to 5-year-old children, the NTP allowed the children in the TG to move to the next developmental step, that is, to form and use an allocentric representation of space.

The TDR of a pathway (in both the L-WalCT and WL-WalCT) did not show an effect of time or training. It must be noted that performances on these tasks were subject to a ceiling effect by the T_0_. This result suggests that once a pathway is learned, its mental representation is stable and long-lasting. This finding is also in line with the performances of young adults in the same task (Piccardi et al., 2008, 2011, 2013).

Moreover, in landmark recognition, we did not identify an effect of time or NTP. We assume that this step is fully developed in 4- to 5-year-old children. It is a first step of environmental knowledge (Siegel and White, 1975) and includes the identification of landmarks (Thorndyke and Hayes-Roth, 1982). This finding is also in line with that of a previous study demonstrating that young individuals unfamiliar with the environment were able to identify landmarks but could not place them on a map (Nori and Piccardi, 2011). Landmark recognition is the first step in the familiarization process with a new environment (Nori and Piccardi, 2011). Interestingly, as opposed to landmark recognition, we found that children improve on the TL of the L-WalCT between T_0_ and T_1_. This result suggests that even if landmark recognition was fully achieved, the ability to integrate landmark-based knowledge with route-based knowledge in the L-WalCT is still developing at age 5. It must be noted that integrating landmark-based knowledge with route-based knowledge is a next step in the normal development of navigational skills. Navigation is typically expected to be easier when landmarks are available in the environment (Nico et al., 2008). Moreover, environments enriched in landmarks assist 5- to 7-year-old children in orienting themselves (Hermer-Vazquez et al., 2001), with an automatic shift toward a landmark-based strategy that results in a significant improvement in performances. This observation also holds in adulthood (Nico et al., 2008; Piccardi, 2009). However, a 20-year-old woman who has never been able to orient herself within the environment as a result of a congenital brain malformation showed the worst performance in a way-finding task when landmarks were available, despite preserved landmark identification (Iaria et al., 2005). This observation also holds in the case of acquired deficits, specifically in right-brain-damaged patients with hemineglect, who did not improve their performances when landmarks were available in the environment (Nico et al., 2008), even if patient performances may be dissociated (Pizzamiglio et al., 1998; Piccardi, 2009). Thus, our data suggest that this developmental step, which may be selectively prevented by a congenital malformation and damaged in acquired brain lesions, is still developing in 4- to 5-year-old children, despite evidence from typical development that demonstrates an earlier acquisition of this step at ~24 months of age (Hermer and Spelke, 1994, 1996).

In general, our results suggest that the normal development of spatial orientation abilities may be considered to follow a continuum rather than a serial organization of navigational mechanisms, which is consistent with previous neuropsychological evidence (e.g., Bianchini et al., 2010, 2014a; Palermo et al., 2014). In light of Siegel and White's cumulative model (1975), we speculate that intermediate stages exist between landmark- and route-based knowledge and between route- and survey-based knowledge. Within these intermediate stages, information coded in different formats is integrated and results in improved performance. Within this framework, even if in the presence of spontaneous improvements in integrating landmark- and route-based knowledge (performance on L-WalCT) and integrating route- and survey-based knowledge (path line drawing), NTP specifically improves performances at the highest level of navigational task. Specifically, the allocentric representation of environmental places arises from the transformation of an egocentric frame of reference. Interestingly, NTP may be useful not only in preventing developmental navigational deficits but also in helping children with visual impairments. As demonstrated by Iossifova and Marmolejo-Ramos (2013), blind children show a difficulty in the use of allocentric coding that may be addressed with specific training, such as an adapted version of NTP for visually impaired children.

Although our results are novel and the future applications in education are interesting, the present study has several weaknesses. In particular, the sample size is small, which represents a potential limitation in the statistical approach that may be adopted. In a future study, we must implement non-parametric or robust approaches to data analyses by increasing the sample size and enrolling participants at different ages of development. Moreover, further studies are needed to understand the long-term effects of the improvement identified in the TG and whether formal training later in life has the same effect on spatial skills. The consequences of NTP in preventing the development of navigational disorders should also be investigated in future studies.

In conclusion, our findings support the idea that the inclusion of formal training of spatial orientation ability during childhood may result in enhanced navigational abilities, particularly for the highest level navigational task.

## Author contributions

LPi, CG, and SD designed and conceived the study. MR and FV collected data. MB, AT, and LPa analyzed data and MB wrote a first draft of the manuscript which has been further revised by all the authors. All the authors contributed to the discussion of the results.

### Conflict of interest statement

The authors declare that the research was conducted in the absence of any commercial or financial relationships that could be construed as a potential conflict of interest.

## Supplementary material

Supplementary Figure 1 **(A)** Experimental timeline; **(B)** An example of an item of the Colored Matrix. The children were instructed to observe the colored boxes in the matrix (up). After 1min of observation, the children were required to turn the page and fill-in the same boxes in an empty matrix (down); **(C)** An example of an item of an unambiguous Paper-and-Pencil Labyrinth. The children were required to help the bee to reach flowers; **(D)** An example of an item of the Objects' Mental Rotation. The children's task was to observe the target flower in the center of the sheet and to identify the only correct one of the three rotated flowers corresponding to the target.
