# Supplementary figures and images for: Enhancing Allocentric Spatial Recall in Pre-schoolers through Navigational Training Programme
Source: Front Neurosci. 2017 Oct 16;11:574. doi: 10.3389/fnins.2017.00574 (PMC5650605; doi:10.3389/fnins.2017.00574)

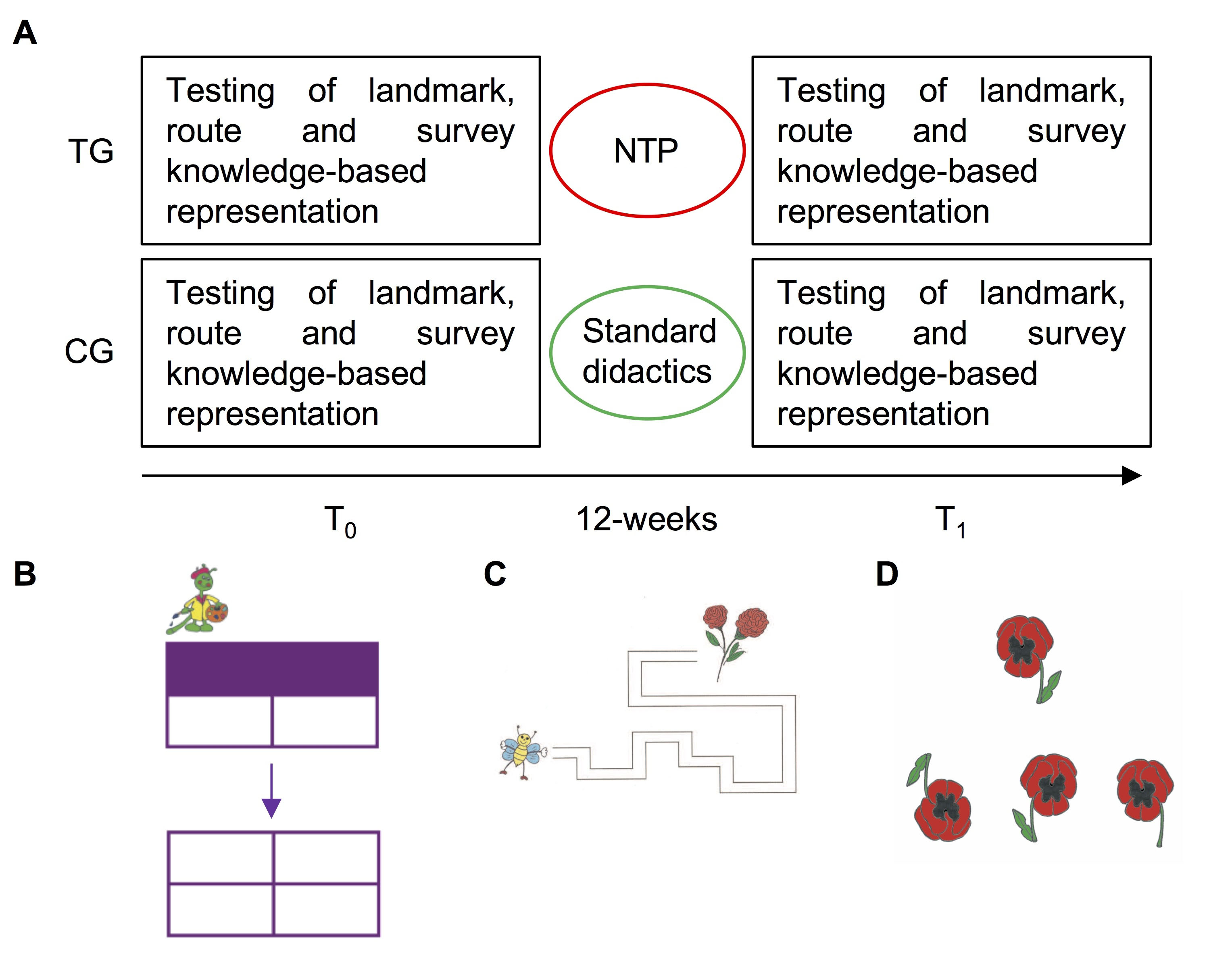

Supplement: Supplementary Figure 1 — (A) Experimental timeline; (B) An example of an item of the Colored Matrix. The children were instructed to observe the colored boxes in the matrix (up). After 1min of observation, the children were required to turn the page and fill-in the same boxes in an empty matrix (down); (C) An example of an item of an unambiguous Paper-and-Pencil Labyrinth. The children were required to help the bee to reach flowers; (D) An example of an item of the Objects' Mental Rotation. The children's task was to observe the target flower in the center of the sheet and to identify the only correct one of the three rotated flowers corresponding to the target. [file Image1.JPEG]
